# Fu’s subcutaneous needling for knee osteoarthritis: a systematic review and meta-analysis

**DOI:** 10.3389/fmed.2025.1602699

**Published:** 2025-08-04

**Authors:** Xiaohu Zhao, Jingxuan Liu, Dake Li, Shangkun Si, Xuanhe Tian, Deke Zhang, Ping Jiang

**Affiliations:** ^1^The First Clinical Medical College, Shandong University of Traditional Chinese Medicine, Jinan, China; ^2^Department of Rheumatology, Affiliated Hospital of Shandong University of Traditional Chinese Medicine, Jinan, China; ^3^Department of Acupuncture, First Teaching Hospital of Tianjin University of Traditional Chinese Medicine, Tianjin, China; ^4^Department of Pharmacy, Affiliated Hospital of Shandong University of Traditional Chinese Medicine, Jinan, China

**Keywords:** acupuncture, Fu’s subcutaneous needling, meta-analysis, osteoarthritis, randomized controlled trials

## Abstract

**Background:**

Acupuncture has been listed as an alternative treatment in several knee osteoarthritis (KOA) international guidelines. Fu’s subcutaneous needling (FSN), as a novel acupuncture therapy, has shown greater potential for treating KOA. The objective of this systematic review is to compare the efficacy and safety of FSN to routine acupuncture therapy (RAT) for KOA.

**Methods:**

China National Knowledge Infrastructure, VIP, China Biomedical Literature Database, Wanfang Medical, Embase, PubMed, Ovid, and the Cochrane Library were searched from inception to March 2025, and randomized controlled trials on FSN for KOA were included. The primary outcomes were total efficacy rate, Visual Analog Scale (VAS) pain scores and Western Ontario and McMaster Universities Arthritis Index (WOMAC) scores. Literature quality was assessed using Cochrane risk-of-bias tool 1.0. Heterogeneity among trials was assessed using the Cochrane *Q* test and *I*^2^ values, determining model selection (fixed/random effects). The meta-analyses of included studies used odds ratios and mean differences when appropriate, along with significance threshold *α* = 0.1. The evidence was evaluated by the GRADE guideline. The PROSPERO International Prospective Register of Systematic Reviews received this research for registration (CRD42024595903).

**Results:**

A total of 14 studies were included (1,186 patients, with 594 in FSN group and 592 in RAT group). Primary outcomes: The total efficacy rate of the FSN group was significantly higher than that of the RAT group [OR = 3.83, 95% CI (2.36, 6.91), *p* < 0.01, *n* = 10, 470/467 participants]. FSN also demonstrated greater effectiveness in reducing VAS pain scores [MD = −1.44, 95% CI (−1.62, −1.26), *p* < 0.01, *n* = 6, 205/206 participants] and WOMAC scores [MD = −6.07, 95% CI (−8.16, −3.97), *p* < 0.01, *n* = 5, 160/161 participants]. Secondary outcomes: FSN group showed a greater reduction in inflammatory cytokines: IL-6 [MD = −1.50 ng/mL, 95% CI (−1.55, −1.46), *p* < 0.01, *n* = 4, 180/180 participants], TNF-α [MD = −2.26 pg/mL, 95% CI (−2.30, −2.23), *p* < 0.01, *n* = 4, 180/180 participants].

**Conclusion:**

Compared to RAT for KOA, FSN demonstrates superior efficacy in alleviating pain, reducing inflammation, and improving joint dysfunction. Further high-quality studies are needed to determine the long-term efficacy of FSN.

**Systematic review registration:**

https://www.crd.york.ac.uk/PROSPERO/view/CRD42024595903.

## 1 Introduction

Knee osteoarthritis (KOA) is a chronic degenerative disease characterized by the progressive destruction of articular cartilage. It often causes persistent pain, limited mobility, and even affects the entire joint, including bone, synovium and joint capsule (1). Epidemiological surveys indicate that the global prevalence of KOA in individuals over the age of 40 is 22.9% (2). Between 1990 to 2019, this number increased by 48% (3). Currently, KOA affects approximately 100 million people worldwide, accounting for 2.2% of global disease burden and ranking as the fourth leading cause of disability (4). Women show a higher prevalence of KOA and greater years lived with disability compared to men, potentially due to anatomical differences influencing knee kinematics (5, 6). The current first-line pharmacological treatment of KOA involves the use of oral non-steroidal anti-inflammatory drugs (NSAIDs), with education, exercise therapy, and weight loss serving as additional primary treatments. Persistent pain is typically managed through oral medications and intra-articular injections, while advanced stages may require surgical interventions, such as joint replacement (7). However, long-term administration of NSAIDs is not recommended due to associated gastrointestinal side effects. Surgical procedures also carry inherent risks, such as implant loosening (8). These limitations highlight the critical need to identify alternative strategies for the prevention and treatment of KOA in clinical practice. Acupuncture is widely recognized for its effectiveness in alleviating pain and improving joint mobility, making it a valuable complementary therapy for KOA. The clinical practice guidelines for KOA in China recommend transcutaneous electrical nerve stimulation (TENS) as an auxiliary rehabilitation intervention following exercise therapy, and these guidelines also advocate for alternative therapy protocols, including manual acupuncture (MA) or electroacupuncture (EA), typically administered over a duration of 4 to 8 weeks (9, 10).

Fu’s subcutaneous needling (FSN), a derivative therapy developed by Dr. Zhonghua Fu based on acupuncture techniques, is characterized by its simplicity, minimal needle insertion, lack of discomfort, and compatibility with patients’ daily activities (11). During the FSN procedure, the needle tip is directed toward the affected muscle, penetrating the subcutaneous layer, followed by sweeping and reperfusion maneuvers (12). Clinical and basic studies have suggested that FSN can reduce quadriceps muscle stiffness. Additionally, FSN has been shown to alleviate nerve injury by inhibiting inflammation and endoplasmic reticulum stress, as well as enhancing mitochondrial structure and energy systems. These effects promote muscle energy metabolism and effectively alleviate pain (13–15). As FSN continues to advance, clinical studies on FSN for the treatment of KOA have increased significantly. However, most of the existing research is limited by small sample sizes, single-center designs, and inconsistent methodologies, posing challenges to the accurate evaluation of its efficacy. Moreover, the comparative advantages of FSN over routine acupuncture therapies (RAT), such as electroacupuncture (EA), manual acupuncture (MA), and electrical stimulation at specific acupoints, still require further clarification. Therefore, this systematic review objectively evaluated the effects of FSN on KOA, focusing on pain reduction, anti-inflammatory effects, functional improvement, and adverse events. The findings aim to provide robust evidence supporting FSN as an emerging non-pharmacological therapy for KOA treatment.

## 2 Materials and methods

### 2.1 Protocol and registration

This systematic review and meta-analysis was reported in accordance with the Preferred Reporting Items for Systematic Reviews and Meta-Analyses (PRISMA) statement, and the PRISMA 2020 Checklists was shown in Supplementary Table S1 (16). This study was registered with international prospective register of systematic reviews (Registration Number: CRD42024595903, Registration Date: 26/11/2024).

### 2.2 Search strategy

A comprehensive search was performed across multiple databases, including China National Knowledge Infrastructure, VIP Database, China Biomedical Literature Database, Wanfang Medical, Embase, PubMed, Ovid, and The Cochrane Library, covering studies published from the inception of each database to March 2025. The search terms included “Fu’s subcutaneous needling,” “Knee Osteoarthritis,” “Osteoarthritis of the Knee,” “Knee Osteoarthritides,” and “Randomized controlled trial.” Details of the search strategy are provided in Supplementary Table S2.

### 2.3 Eligibility criteria

Inclusion criteria include (a) Population: KOA patients, no restrictions on age, gender, or nationality. (b) Interventions: FSN group only received FSN treatment (needling near the myofascial trigger points of the affected muscles, with the number of points not fixed). (c) Comparator: The control group received RAT, including but not limited to MA, EA, and TENS in combination with specific acupoints, also known as transcutaneous electrical acupoint stimulation (TEAS), with the number of acupoints not fixed. (d) Outcomes: The primary outcomes included the total effective rate and pain intensity scales (such as Visual Analog Scale, VAS pain scores) and knee function scales (Western Ontario and McMaster Universities Arthritis Index, WOMAC scores). Secondary outcomes focused on improvements in inflammatory cytokines levels in synovial fluid (such as IL-6 and TNF-α) and adverse events associated with the interventions. The pain intensity scale and the knee function scale used in more than two included studies were used as outcome indicators. (e) Study type: RCTs, no language restrictions.

Exclusion criteria include (a) Other arthritis patients. (b) Studies in which FSN was combined with another intervention or compared to placebo. (c) Literature with incomplete outcome indicators or original articles could not be obtained.

### 2.4 Data extraction and quality assessment

NoteExpress (17) was used to compile, remove duplication, screen literature and extract research data. Two independent reviewers conducted an initial review based on titles and abstracts, followed by full-text screening. Agreement between the independent reviewers was assessed using Cohen’s kappa statistic (18). Any disagreements were submitted to a designated corresponding author for arbitration. Finally, data such as country of study, authors’ names, publication years, funding sources for the studies, sample sizes, interventions, grade of KOA, baseline pain/function scores, treatment duration/follow-up, and outcome indicators were extracted.

The quality evaluation of the included studies was based on the Cochrane Handbook for Systematic Reviews 5.1 and the Risk of Bias Assessment Tool 1.0 (19). The evaluation focused on the following criteria: “Generation of randomisation,” “allocation concealment,” “blinding of investigator subjects,” “blinding of outcome measures,” “outcome data integrity and selection reporting bias,” and “other sources of bias.” Two researchers independently conducted the quality assessment. Any disagreements were submitted to a designated corresponding author for arbitration.

The quality of evidence was assessed using the GRADE (Grading of Recommendations, Assessment, Development, and Evaluations) framework, and the following areas were included: indirectness, imprecision, risk of bias, inconsistency, publication bias, and other considerations. The quality of evidence was categorized as “high,” “moderate,” “low,” or “very low” quality (20). Specific evaluation rules are listed in the Supplementary Appendix S1.

### 2.5 Statistical analysis

RevMan 5.3 software (21) was used for statistical analysis. Continuous variables were described by mean difference (MD) with 95% confidence interval (CI). Dichotomous variables were statistically analyzed with odds ratio (OR) and 95% CI. Heterogeneity among studies was assessed using the chi-square test with *α* = 0.1, and the degree of heterogeneity was evaluated based on the *I*^2^ value. This study reported *I*^2^ using the following general rules: 0 to 25% represents low heterogeneity, 26 to 50% represents moderate heterogeneity, and 51 to 75% represents significant heterogeneity (22). If *p* ≥ 0.1 and *I*^2^ ≤ 50%, this indicates good homogeneity among the included studies, then a fixed effect model was used for meta-analysis. If *p* < 0.1 and *I*^2^ > 50%, this indicates significant heterogeneity among the included studies, then a random effects model was used, and then a subgroup analysis or sensitivity analysis was performed to find the source of heterogeneity. When there was a large proportion term, sensitivity analysis was used to check the stability of the results. If a sufficient number of articles were, the risk of publication bias was evaluated visually using funnel plots and with Egger’s test (Stata 17.0), considering a *p*-value <0.1 as indicative of significant publication bias (23).

## 3 Results

### 3.1 Literature search

**Figure 1:**
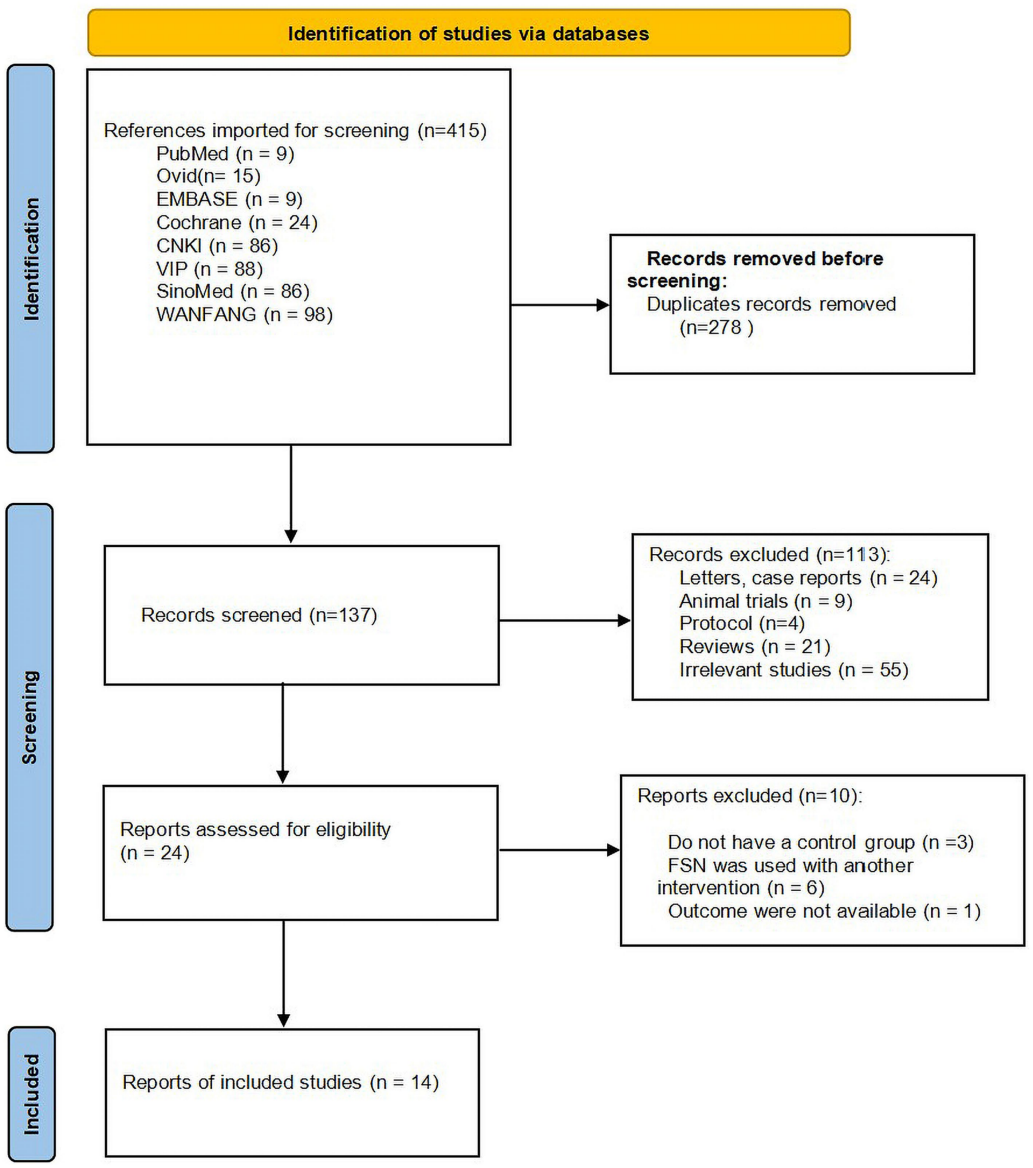
PRISMA flow diagram.

According to the search strategy, 415 articles were obtained, and 278 articles were retrieved after eliminating duplicates. Both independent reviewers applied the exclusion criteria and excluded 113 articles that were not relevant to this review, with inter-reviewer reliability (*K* = 0.807) based on the kappa statistic. Twenty-four articles were potentially relevant to this study and selected for full-text evaluation. Of the 24 full-text articles evaluated, 10 articles (24–33) were excluded with the inter-reviewer reliability (*K* = 1) based on the kappa statistic. Ultimately, 14 studies that meet the inclusion criteria were included, involving a total of 1,186 patients, with 594 in FSN group and 592 in RAT group. Supplementary Table S3 lists the excluded studies and reasons for exclusion. The literature screening process is illustrated in Figure 1, and the basic characteristics of the included studies are summarized in Table 1.

**Table 1:** Study characteristics.

Country of study	Authors, year	Age mean (SD)		Sample size (male/female)		Intervention		Grade of KOA	Baseline pain (VAS)/Function scores (WOMAC)		FSN therapy	Treatment duration/Follow-up	Outcomes
		T	C	T	C	T	C		T	C			
China	Wang et al., 2024 (34)	55 (9)	55 (8)	8/22	7/23	FSN	EA	Early and moderate stage	4.47 (0.9)/—	4.47 (1.14)/—	Once every other day, 5 times in a row	10 days/3 months	PI
China	Mou et al., 2024 (35)	54.87 (8.85)	55.10 (7.98)	8/22	7/23	FSN	EA	Early and moderate stage	—	—	Once every other day, 5 times in a row	10 days/3 months	ADs
China	Chen, 2023 (36)	55.4 (5.5)	55.25 (5.46)	18/22	17/23	FSN	EA	Kellgren–Lawrence grade (I–III)	7.52 (1.2)/—	7.46 (1.12)/—	Once every other day, 5 consecutive times is a course of treatment, with a total of 3 courses of treatment.	One month/—	PI, LICSF
China	Sheng et al., 2022 (37)	60.03 (8.25)	60.69 (6.55)	8/27	10/25	FSN	EA	Early and moderate stage	—/44.77 (10.90)	—/45.77 (13.17)	Once every other day, 3 times a week, for a total of 2 weeks	2 weeks/2 months	TER, KF ADs
China (Taiwan)	Chiu et al., 2022 (13)	65.73 (6.79)	62.81 (5.72)	4/11	6/10	FSN	TEAS	Kellgren–Lawrence grade (II~)	5.8 (1.42)/27.8 (12.12)	5.81 (0.91)/26.44 (12.54)	Once every other day, 3 times a week, for a total of 2 weeks	2 weeks/14 days	PI, KF
China	Liu et al., 2020 (38)	65 (10)	58 (13)	26/14	22/18	FSN	MA	—	—/69.7 (6.5)	—/69.1 (7.3)	Once every other day, 3 times a week, for a total of 2 weeks	2 weeks/—	TER, KF
China	Huang et al., 2020 (39)	52.21 (8.65)	52.52 (9.3)	18/23	20/30	FSN	EA	Kellgren–Lawrence grade (I–III)	7.69 (2.21)/—	1.38 (1.26)/—	Once every other day, 3 times a week, for a total of 2 weeks	2 weeks/—	TER, PI LICSF
China	Zhang et al., 2020 (40)	56.25 (3.73)	55.74 (3.62)	11/29	8/32	FSN	MA	—	5.71 (0.54)/49.76 (7.41)	5.89 (0.67)/50.13 (7.32)	Once every other day, 3 times a week, for a total of 2 weeks	2 weeks/—	TER, PI KF
China	Zhu, 2019 (41)	62.17 (8.46)	63.14 (8.12)	18/20	17/21	FSN	EA	—	—	—	Once every other day, 5 times in a row	10 days/—	TER LICSF
China	Li et al., 2018 (42)	56.33 (7.59)	55.87 (7.29)	7/23	8/22	FSN	MA	Kellgren-Lawrence grade (I–III)	5.9 (0.76)/31.1 (9.15)	6.03 (0.89)/33.47 (11.36)	Once every other day, 3 times a week, for a total of 2 weeks	14 days/one month	TER, PI KF
China	Feng et al., 2017 (44)	55.67 (4.98)	54.25 (4.36)	29/31	28/32	FSN	EA	Kellgren–Lawrence grade (I–III)	—	—	Once every other day, 5 times in a row	10 days/—	TER LICSF
China	Li, 2016 (45)	52	55	14/31	7/35	FSN	MA	—	—	—	Once every other day, 5 consecutive times is a course of treatment, with a total of 2 courses of treatment	10 days/—	TER
China	Liu et al., 2013 (43)	52.8 (3.2)	53.6 (2.7)	23/27	24/26	FSN	EA	—	—	—	Once every other day, 5 consecutive times is a course of treatment, with a total of 3 courses of treatment	2 weeks/—	TER
China	Wang et al., 2011 (46)	45–88	48–85	30/60	28/62	FSN	EA	—	—	—	Once every other day, 5 times in a row	10 days/—	TER

FSN, Fu’s subcutaneous needling; EA, electroacupuncture; TENS, transcutaneous electrical nerve stimulation; MA, manual acupuncture; TCM, traditional Chinese medicine; T, trial group; C, control group; TER, total efficacy rate; PI, pain intensity (VAS pain scores); KF, knee function (WOMAC scores); LICSF, levels of inflammatory cytokines in synovial fluid (IL-6, ng/mL and TNF-α, pg/mL), ADs, adverse events.

### 3.2 Quality assessment of included studies

**Figure 2:**
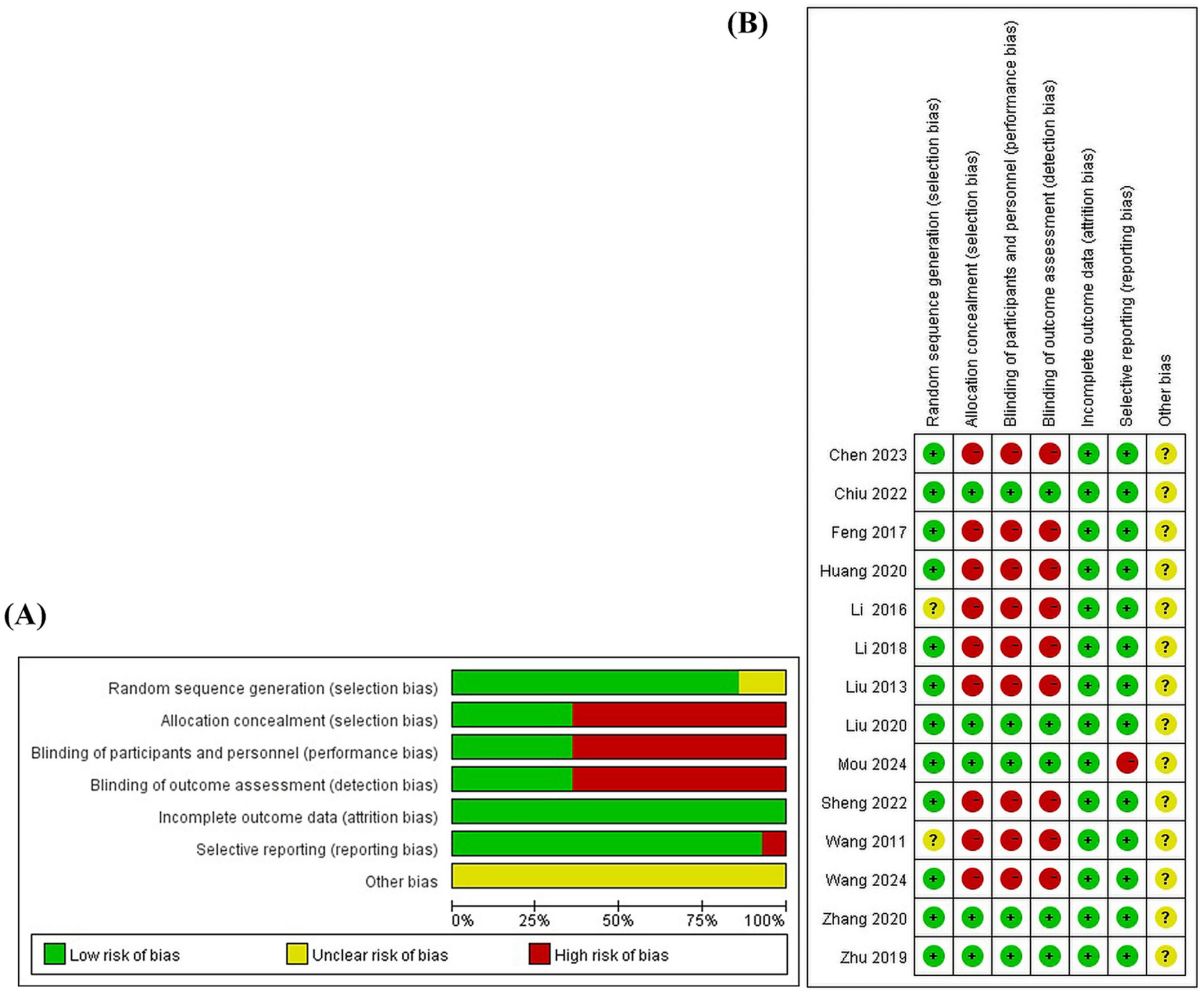
The risk of bias of the included studies. **(A)** Risk of bias graph. **(B)** Risk of bias summary.

Two independent researchers evaluated 14 articles using the Bias Risk Assessment Tool 1.0, with the inter-reviewer reliability based on the kappa statistic. Random sequence generation: All 14 included articles mentioned the use of random allocation, 11 of which used a random number table method (13, 34–43), and one used coin tossing (44), so the risk of bias was considered low risk. Two articles only mentioned randomization without describing how random sequence was decided (45, 46), so the risk of bias was considered uncertain. Allocation concealment: five articles used sealed envelopes for allocation concealment (13, 35, 38, 40, 41), so the risk of bias was considered low. While the rest did not mention randomization, the risk of bias was considered high. Blinding of participants and personal: five articles (13, 35, 38, 40, 41) reported single-blinding for patients (as single-blinding is the only feasible option in acupuncture studies due to the inability to blind practitioners) and blinded outcome assessment, so the risk of bias was considered low. The remaining studies did not report blinding, so the risk of bias were considered high. Blinding of outcome assessment: five articles (13, 35, 38, 40, 41) illustrated blinding of outcome assessments, so the risk of bias was considered low. The remaining studies did not specify the blinding of outcome assessment and were considered to have a high risk of bias. Incomplete outcome data: None of the included studies had missing data for the outcomes, so the risk of bias was low. Selective reporting: All studies, except one (35), reported at least one desired primary outcome, so it was considered to be at a high risk of bias, and all other studies were at a low risk of bias. Other bias: 14 studies had insufficient information to assess whether they presented an important risk of bias, so the risk of bias were considered uncertain. The quality of the studies was evaluated using the Cochrane risk of bias assessment tool (Figure 2), and the results showed that the overall quality was fair.

### 3.3 Primary outcome indicators

#### 3.3.1 Total efficacy rate

**Figure 3:**
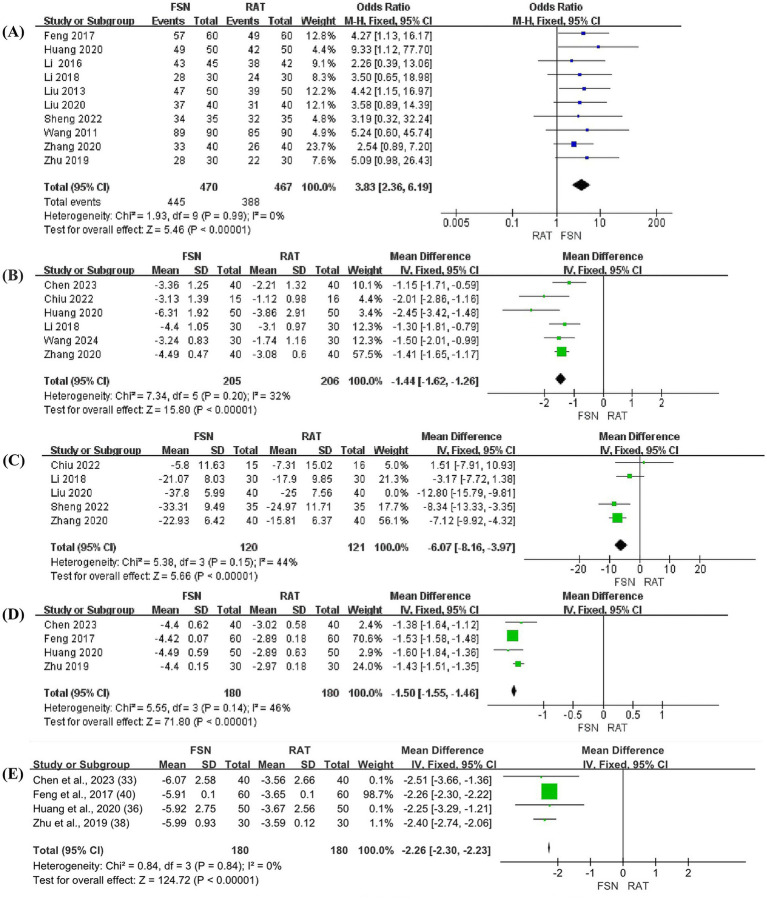
The forest plots (FSN vs. RAT). **(A)** Total efficacy rate. **(B)** VAS pain scores. **(C)** The total score of WOMAC. **(D)** The level of IL-6 in synovial fluid. **(E)** The level of TNF-α in synovial fluid.

Ten studies reported the total efficacy rate of FSN group compared to RAT group in the prevention and treatment of KOA, involving 937 patients (470 in FSN group and 467 in RAT group). Heterogeneity test (*p* = 0.99, *I*^2^ = 0%) showed no significant statistical heterogeneity, indicating low sensitivity and good stability. A fixed effect model was used to combine the effect size. The results showed that the total efficacy rate of FSN group was significantly higher than that of RAT group [OR = 3.83, 95% CI (2.36, 6.91), *p* < 0.01], as shown in Figure 3A.

#### 3.3.2 VAS pain scores

Six studies reported the effect of FSN compared to RAT in reducing VAS pain scores for KOA patients, involving 411 patients (205 in FSN group and 206 in RAT group). Heterogeneity test (*p* = 0.20, *I*^2^ = 32%) showed no significant statistical heterogeneity, indicating low sensitivity and good stability. A fixed effect model was used to combine effect size. The results suggested that FSN group was more effective than RAT group in reducing VAS pain scores of KOA patients [MD = −1.44, 95% CI (−1.62, −1.26), *p* < 0.01], as shown in Figure 3B.

#### 3.3.3 The total scores of WOMAC

Five studies reported the effect of FSN compared to RAT in reducing the total score of WOMAC for patients with KOA, involving 321 patients (160 in FSN group and 161 in RAT group). A heterogeneity test was initially performed (*p* = 0.001, *I*^2^ = 78%), which showed significant heterogeneity. A sensitivity analysis was then conducted, and one study was excluded. After excluding this study, the heterogeneity test (*p* = 0.15, *I*^2^ = 44%). No significant statistical heterogeneity was shown, indicating low heterogeneity and good stability. Using a fixed effect model to combine effect size, the results suggested that FSN group was more effective than RAT group in reducing WOMAC scores of KOA patients [MD = −6.07, 95% CI (−8.16, −3.97), *p* < 0.01], as shown in Figure 3C.

### 3.4 Secondary outcome indicators

#### 3.4.1 Levels of inflammatory cytokines (IL-6, TNF-α) in synovial fluid

Four studies reported the effect of FSN compared to RAT on reducing the inflammatory cytokines levels (IL-6 and TNF-α) in synovial fluid of KOA joint, involving 360 patients (180 in FSN group and 180 in RAT group).

For IL-6, the heterogeneity test (*p* = 0.14, *I*^2^ = 46%) showed no significant statistical heterogeneity, indicating low sensitivity and good stability. A fixed effect model was used to combine the effect size. The results suggested that FSN group was more effective than RAT group in reducing the level of IL-6 in the synovial fluid of KOA patients [MD = −1.50, 95% CI (−1.55, −1.46), *p* < 0.01], as shown in Figure 3D.

For TNF-α, the heterogeneity test (*p* = 0.84, *I*^2^ = 0%) showed no significant statistical heterogeneity, indicating low sensitivity and good stability. A fixed effect model was used to combine the effect size. The results suggested that FSN group was more effective than RAT group in reducing the level of TNF-α in the synovial fluid of KOA patients [MD = −2.26, 95% CI (−2.30, −2.23), *p* < 0.01], as shown in Figure 3E.

#### 3.4.2 Adverse events

Only two studies reported the number of adverse events. Due to the insufficient number of studies (less than three), we decided not to perform meta-analysis but rather conducted descriptive analysis only. The overall incidence rates of adverse events in these two studies were 6.5 and 5.7%, respectively.

In Study 1 (35), the incidence rates of adverse events were 3.2% in the FSN group versus 9.7% in the RAT group. Similarly, Study 2 (37) showed incidence rates of 2.9% in the FSN group compared with 8.6% in the RAT group.

### 3.5 Publication bias

**Figure 4:**
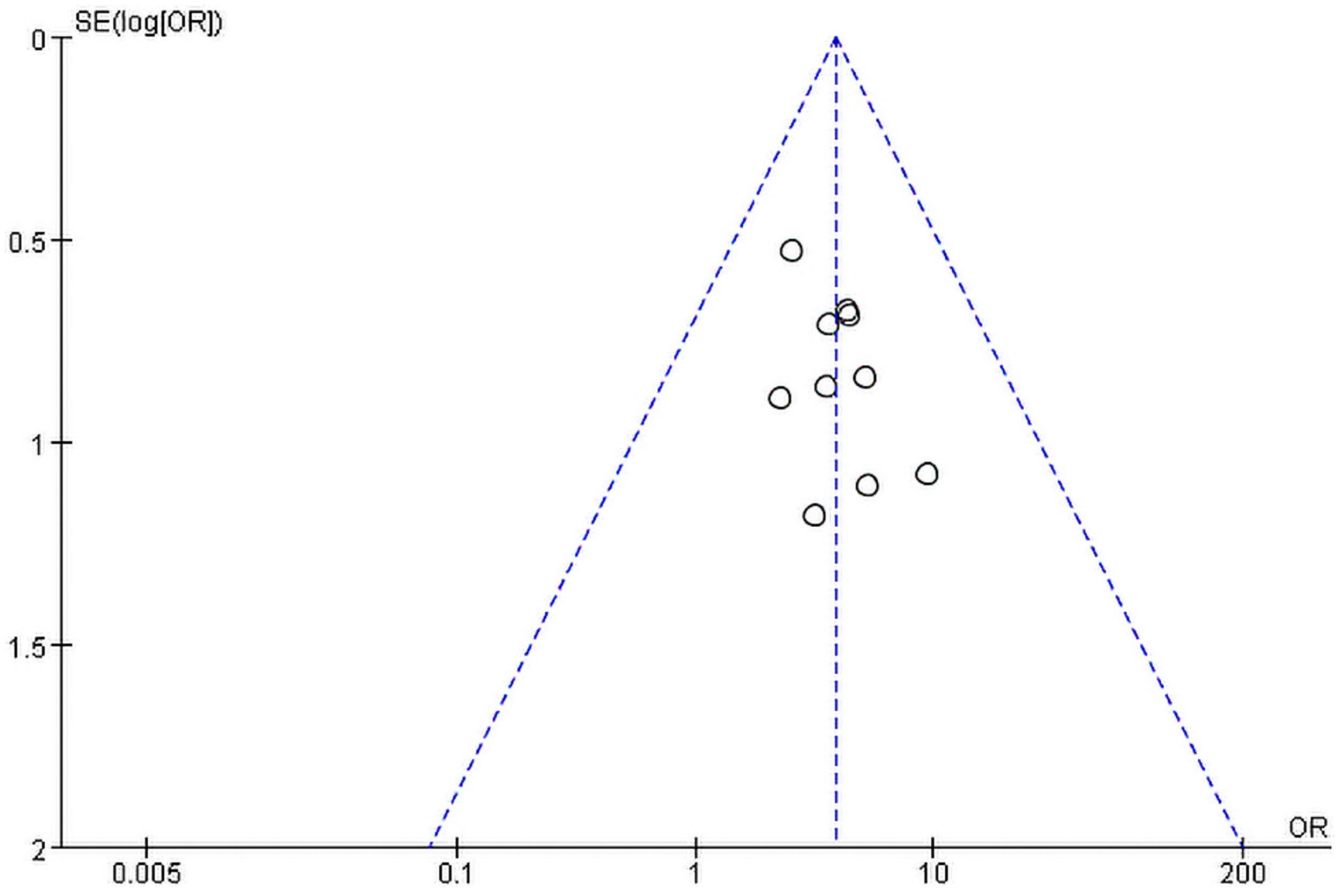
The funnel plot of the total efficacy rate.

Revman5.3 software was used to create the funnel plot for “Total efficacy rate.” The diagram (Figure 4) showed good bilateral symmetry along the central axis, with no apparent publication bias. The Egger’s test results also indicated no significant statistical publication bias (*p* = 0.192). For the other meta-analyses, no funnel plot was create due to an insufficient number of included studies. Only Egger’s test was used to assess publication bias, and no significant publication bias was found (*p* > 0.1). The results of Egger’s test were shown in Supplementary Appendix S2.

### 3.6 GRADE evidence quality classification

The quality of evidence for all outcomes was evaluated using the GRADE guidelines. Due to methodological limitations, five outcome indicators (Total efficacy rate, VAS pain score, WOMAC score, IL-6, and TNF-α) were assessed as having moderate quality. The GRADE evidence profiles and summary of findings table were shown in Table 2.

**Table 2:** GRADE evidence profiles and summary of findings table.

Outcome (studies)	No of participants		Risk of bias	Inconsistency	Indirectness	Imprecision	Other considerations	Overall certainty of evidence	Anticipated absolute effects (95% CI)
	FSN	RAT							
Total efficacy rate (10 RCTs)	470	467	Serious^a^	Not serious	Not serious	Not serious	None	⨁⨁⨁◯ Moderate	OR 3.83 lower (2.36 lower to 6.19 lower)
VAS pain scores (6 RCTs)	205	206	Serious^a^	Not serious	Not serious	Not serious	None	⨁⨁⨁◯ Moderate	MD −1.44 lower (−1.62 lower to −1.26 lower)
WOMAC scores (5 RCTs)	120	121	Serious^a^	Not serious	Not serious	Not serious	None	⨁⨁⨁◯ Moderate	MD −6.07 lower (−8.16 lower to −3.97 lower)
IL-6 (ng/mL) (4 RCTs)	180	180	Serious^a^	Not serious	Not serious	Not serious	None	⨁⨁⨁◯ Moderate	MD −1.50 lower (−1.55 lower to −1.46 lower)
TNF-α (pg/mL) (4 RCTs)	180	180	Serious^a^	Not serious	Not serious	Not serious	None	⨁⨁⨁◯ Moderate	MD −2.26 lower (−2.30 lower to −2.23 lower)

FSN, Fu’s subcutaneous needling; RAT, routine acupuncture therapy; VAS, Visual Analog Scale; WOMAC, Western Ontario and McMaster Universities Arthritis Index.

a Half of the studies lacked allocation concealment methods, and blinded evaluation.

## 4 Discussion

### 4.1 Summary of the main findings

This study suggests that, compared to the RAT group, patients with KOA in the FSN group had higher total efficacy after the treatment, with lower VAS pain scores and WOMAC scores. These results indicate that FSN may help control pain symptoms and restore knee function in KOA patients. The FSN group also exhibited lower levels of synovial inflammatory cytokines (IL-6, TNF-α), suggesting that FSN may help control inflammation-related joint symptoms in KOA patients. However, only two studies reported adverse events, and this meta-analysis merely conducted a descriptive analysis. The safety comparison between FSN and RAT requires further investigation.

### 4.2 Clinical basis and therapeutic mechanism of FSN

Decreases in VAS pain scores and WOMAC scores indicate that FSN improves pain and modulates joint function in KOA patients. The vascular channels in articular cartilage contain sensory nerve endings, and the related neural innervation may contribute to KOA pain (47). FSN may promote tissue repair in the lesion microenvironment by facilitating nerve growth and increasing myelin regeneration factors, thereby modulating nerve injury (14), which could be associated with alleviation of neural pain and improvement in joint function. The primary characteristics of KOA are related to cartilage damage, with a vicious cycle of chondrocyte activity leading to the activation of inflammatory pathways (48). The inflammatory cytokines IL-6 and TNF-α are considered the main pro-inflammatory mediators in KOA (49). FSN treatment has been shown to improve skeletal muscle mitochondrial function and increase tissue permeability, thereby promoting the metabolism of inflammatory factors and exerting anti-inflammatory effects (15). Furthermore, because inflammatory cytokines are involved in pain modulation (50), the anti-inflammatory effects of FSN may also contribute to pain relief. Overall, FSN alleviates pain by stimulating the loose connective tissue beneath the skin, which contains collagen with liquid crystalline structures and piezoelectric properties. When the needle moves within this subcutaneous tissue, bioelectrical signals are generated. These signals trigger a reverse piezoelectric effect upon reaching the injured tissues, modulating ion channels, promoting vasodilation, and inducing local muscle relaxation (51). This process enhances the circulation and metabolism of interstitial fluid and blood (52). For example, a study by Yang et al. (53) found that FSN treatment significantly increased the levels of oxygenated hemoglobin and total hemoglobin in the vastus lateralis muscle of KOA patients. These combined effects are thought to realign the lower limb’s force axis, reduce abnormal stress on the cartilage, and help maintain knee joint stability. Based on the aforementioned mechanism, Huang et al. (54) designed an RCT protocol to evaluate the biomechanical effects of FSN in the treatment of senile KOA. Future clinical data on the biomechanical improvements of FSN in KOA may further elucidate its potential therapeutic mechanisms.

While the mechanistic evidence supports FSN’s efficacy, its comparative effectiveness against established therapies requires further exploration. Wang et al. (55) compared the effects of non-pharmacological interventions with NSAIDs on VAS, WOMAC scores, and inflammatory cytokine levels in KOA patients, finding that radiofrequency treatment among the non-pharmacological interventions was more effective in improving clinical symptoms. Although our study also confirmed that FSN improves clinical symptoms and exerts anti-inflammatory effects in KOA, there is currently a lack of RCTs directly comparing FSN with NSAIDs and other alternative treatments recommended in clinical guidelines (e.g., physical therapy and muscle function training). This suggests that future studies could explore whether FSN represents a superior treatment option (56, 57).

Gregori et al. (58) conducted a systematic review using WOMAC and VAS scores to evaluate the long-term outcomes of drug treatment for KOA pain. They found that when the trial duration exceeded 12 months, most drugs lacked sufficient evidence of a correlation with pain improvement, with the exception of glucosamine sulfate. In contrast, our results demonstrate that FSN can significantly reduce VAS pain scores and WOMAC scores in the short term, although its long-term effects have yet to be adequately verified. Eight RCTs (13, 34–38, 42, 44) included in this meta-analysis provided information on the grading of KOA, all of which were classified as early to moderate stage. The remaining 6 RCTs (38, 40, 41, 43, 45, 46) did not provide additional details regarding the grade of KOA. Considering the irreversible progression of bone destruction in late-stage KOA, this study proposes that FSN may be more suitable for early-to-mid-stage KOA, where it can help alleviate pain and correct pathological biomechanical imbalances in the affected knee joint. FSN may not be appropriate for advanced stages of the disease where structural damage is irreversible.

Studies have highlighted the therapeutic advantages of FSN compared to acupuncture modalities. A network meta-analysis evaluating seven acupuncture techniques for treating KOA suggested that FSN ranked second in efficacy, just below silver needle therapy and superior to other methods such as needle-knife therapy, fire needle therapy, and EA. (59) Traditional acupuncture therapies typically target specific acupoints or Ashi points (painful spots or palpation points) for treatment, while other rehabilitation therapies such as dry needling target myofascial trigger points (MTrPs). The concept of Ashi points or MTrPs focuses on localized areas or myofascial tension, and there is difficulty in identifying the precise source of pain (60). In contrast, FSN targets progressive regions from the pain point to the MTrP to the tightened muscle. Pathological tension is predominantly found in the muscle belly and is often not localized to a point but manifests as diffuse, ribbon-like, or columnar areas. The unique advantage of FSN is that pain is no longer regarded as a single point but as a reflection of underlying tissue injury. Combined with swaying motion, FSN can better realign the force axis disorder of knee joint movement caused by peripheral muscle lesions. Additionally, FSN introduces an innovative and simplified procedure: inserting a solid needle into the subcutaneous layer between the skin and muscle, performing a swaying motion, and then applying a flushing technique. After completing the swaying motion, the solid needle is withdrawn, leaving a soft catheter in the subcutaneous layer at the acupuncture site for 8 h (46). Thus, another advantage of FSN lies in its accessibility. Practitioners can quickly learn this technique without requiring in-depth knowledge of traditional Chinese medicine or acupoints, as it relies solely on their existing medical training.

### 4.3 Limitations

The limitations of this study are as follows: (a) Study quality and internal validity: While all included studies mentioned randomization, some failed to specify randomization methods, potentially introducing selection bias. The majority of studies exhibited moderate quality limitations, particularly regarding allocation concealment and blinding procedures, which were either inadequately reported or absent, increasing risks of both selection and measurement bias. Furthermore, the unclear sample size estimation methods in some studies raise concerns about statistical power adequacy. These methodological shortcomings collectively weaken the certainty of our pooled estimates. Additionally, the lack of reported follow-up data in most studies limits our ability to assess long-term efficacy. (b) Inclusion criteria: Only VAS and WOMAC scores were included in this systematic review. Due to the limitations of the outcome indicators reported in the literature, other common knee joint function and pain intensity scores, such as the Lequesne Index, Lysholm Score, and Numeric Rating Scale (NRS), were not analyzed, which may compromise the robustness of the meta-analysis. (c) Intervention: The specific acupuncture methods used in the control groups varied, involving MA, EA and TEAS. Although FSN is considered a standardized therapy, we observed variations in its operational parameters, including the selection of the “tightened muscle,” the number of treatment sessions, and post-needle retention techniques. Similarly, there were differences in the acupoint selection and stimulation intensity during RAT treatment. These variations in treatment protocols across studies represent important limitations and potential sources of heterogeneity. Furthermore, differences in time, location and treatment course across different trial centers might introduce systematic errors. (d) Literature source: All the research centers in this study were located in China, so the lack of supporting evidence from other countries might limit the generalizability of the findings. (e) Others: Unlike pharmacological clinical studies, where dose–response relationships are typically well-defined, non-pharmacological interventions involve multiple factors, making the dose-response relationship of acupuncture interventions in the included studies difficult to clarify.

## 5 Conclusion

In conclusion, current evidence suggests that FSN offers significant benefits for patients with KOA when compared to RAT, particularly in alleviating pain, improving knee joint function, and reducing synovial inflammatory factors. FSN is a simple and promising alternative treatment modality. However, further research is needed to compare its efficacy with other guideline-recommended alternative therapies, such as physical therapy. Additionally, the studies included in this systematic review had small sample sizes, relatively low methodological quality, and limited credibility. Some of the included RCTs did not address specific KOA disease stages or provide follow-up data. Therefore, future FSN researchers should implement global training programs targeting traditional Chinese medicine universities and pain research institutions interested in novel acupuncture therapies and non-pharmacological pain management, while establishing multinational RCT studies to generate robust evidence regarding optimal disease stages for FSN application and long-term therapeutic outcomes in KOA management.

## Data Availability

The original contributions presented in the study are included in the article/Supplementary material, further inquiries can be directed to the corresponding author.
