# Correction: Fu's subcutaneous needling for knee osteoarthritis: a systematic review and meta-analysis

**DOI:** 10.3389/fmed.2025.1686449

**Published:** 2025-09-02

**Authors:** Xiaohu Zhao, Jingxuan Liu, Dake Li, Shangkun Si, Xuanhe Tian, Deke Zhang, Ping Jiang

**Affiliations:** ^1^The First Clinical Medical College, Shandong University of Traditional Chinese Medicine, Jinan, China; ^2^Department of Rheumatology, Affiliated Hospital of Shandong University of Traditional Chinese Medicine, Jinan, China; ^3^Department of Acupuncture, First Teaching Hospital of Tianjin University of Traditional Chinese Medicine, Tianjin, China; ^4^Department of Pharmacy, Affiliated Hospital of Shandong University of Traditional Chinese Medicine, Jinan, China

**Keywords:** acupuncture, Fu's subcutaneous needling, meta-analysis, osteoarthritis, randomized controlled trials

Affiliation 2 was erroneously given as “Department of Rheumatology, Affiliated Hospital of Shandong University of Traditional Chinese Medicine, Yintai, China”. The correct affiliation 2 is “Department of Rheumatology, Affiliated Hospital of Shandong University of Traditional Chinese Medicine, Jinan, China.”

The funder Shandong Provincial Health and Wellness Science and Technology Innovation Team Construction Project, [2024sdskctd-03] to Ping Jiang was erroneously omitted.

Affiliation for Reviewer Kian Djien Liem was erroneously given as “Radboud University, Netherlands”. The correct affiliation is “Foundation for Doctors and Medical Acupuncture (SAMEDA), Netherlands”.

The original version of this article has been updated.

