# Supplementary material for: Fu’s subcutaneous needling for knee osteoarthritis: a systematic review and meta-analysis
Source: Front Med (Lausanne). 2025 Aug 4;12:1602699. doi: 10.3389/fmed.2025.1602699 (PMC12358445; doi:10.3389/fmed.2025.1602699)

***Supplementary Material***

**Supplemental Table S1**. Preferred Reporting Items for Systematic Reviews and Meta-Analyses 2020 (PRISMA 2020) Checklists

PRISMA 2020 Main Checklist

| **Topic** | **No.** | **Item** | **Location where item is reported** |
| --- | --- | --- | --- |
| **TITLE** |  |  |  |
| **Title** | 1 | Identify the report as a systematic review. | Page 1 |
| **ABSTRACT** |  |  |  |
| **Abstract** | 2 | See the PRISMA 2020 for Abstracts checklist. |  |
| **INTRODUCTION** |  |  |  |
| **Rationale** | 3 | Describe the rationale for the review in the context of existing knowledge. | Page 2-3 |
| **Objectives** | 4 | Provide an explicit statement of the objective(s) or question(s) the review addresses. | Page 3 |
| **METHODS** |  |  |  |
| **Eligibility criteria** | 5 | Specify the inclusion and exclusion criteria for the review and how studies were grouped for the syntheses. | Page 4 |
| **Information sources** | 6 | Specify all databases, registers, websites, organisations, reference lists and other sources searched or consulted to identify studies. Specify the date when each source was last searched or consulted. | Page 3, Supplemental Table S2 |
| **Search strategy** | 7 | Present the full search strategies for all databases, registers and websites, including any filters and limits used. | Page 3, Supplemental Table S2 |
| **Selection process** | 8 | Specify the methods used to decide whether a study met the inclusion criteria of the review, including how many reviewers screened each record and each report retrieved, whether they worked independently, and if applicable, details of automation tools used in the process. | Page 4 |
| **Data collection process** | 9 | Specify the methods used to collect data from reports, including how many reviewers collected data from each report, whether they worked independently, any processes for obtaining or confirming data from study investigators, and if applicable, details of automation tools used in the process. | Page 4 |
| **Data items** | 10a | List and define all outcomes for which data were sought. Specify whether all results that were compatible with each outcome domain in each study were sought (e.g. for all measures, time points, analyses), and if not, the methods used to decide which results to collect. | Page 4 |
|  | 10b | List and define all other variables for which data were sought (e.g. participant and intervention characteristics, funding sources). Describe any assumptions made about any missing or unclear information. | Page 4 |
| **Study risk of bias assessment** | 11 | Specify the methods used to assess risk of bias in the included studies, including details of the tool(s) used, how many reviewers assessed each study and whether they worked independently, and if applicable, details of automation tools used in the process. | Page 4 |
| **Effect measures** | 12 | Specify for each outcome the effect measure(s) (e.g. risk ratio, mean difference) used in the synthesis or presentation of results. | Page 5 |
| **Synthesis methods** | 13a | Describe the processes used to decide which studies were eligible for each synthesis (e.g. tabulating the study intervention characteristics and comparing against the planned groups for each synthesis (item 5)). | Page 5 |
|  | 13b | Describe any methods required to prepare the data for presentation or synthesis, such as handling of missing summary statistics, or data conversions. | Page 5 |
|  | 13c | Describe any methods used to tabulate or visually display results of individual studies and syntheses. | Page 5 |
|  | 13d | Describe any methods used to synthesize results and provide a rationale for the choice(s). If meta-analysis was performed, describe the model(s), method(s) to identify the presence and extent of statistical heterogeneity, and software package(s) used. | Page 5 |
|  | 13e | Describe any methods used to explore possible causes of heterogeneity among study results (e.g. subgroup analysis, meta-regression). | Page 5 |
|  | 13f | Describe any sensitivity analyses conducted to assess robustness of the synthesized results. | Page 5 |
| **Reporting bias assessment** | 14 | Describe any methods used to assess risk of bias due to missing results in a synthesis (arising from reporting biases). | Page 5 |
| **Certainty assessment** | 15 | Describe any methods used to assess certainty (or confidence) in the body of evidence for an outcome. | Page 5 |
| **RESULTS** |  |  |  |
| **Study selection** | 16a | Describe the results of the search and selection process, from the number of records identified in the search to the number of studies included in the review, ideally using a flow diagram. | Figure 1 |
|  | 16b | Cite studies that might appear to meet the inclusion criteria, but which were excluded, and explain why they were excluded. | Figure 1  S3 Table |
| **Study characteristics** | 17 | Cite each included study and present its characteristics. | Table 1 |
| **Risk of bias in studies** | 18 | Present assessments of risk of bias for each included study. | Figure 2 |
| **Results of individual studies** | 19 | For all outcomes, present, for each study: (a) summary statistics for each group (where appropriate) and (b) an effect estimate and its precision (e.g. confidence/credible interval), ideally using structured tables or plots. | Figure 3 |
| **Results of syntheses** | 20a | For each synthesis, briefly summarise the characteristics and risk of bias among contributing studies. | Page 9 |
|  | 20b | Present results of all statistical syntheses conducted. If meta-analysis was done, present for each the summary estimate and its precision (e.g. confidence/credible interval) and measures of statistical heterogeneity. If comparing groups, describe the direction of the effect. | Page 9-10 |
|  | 20c | Present results of all investigations of possible causes of heterogeneity among study results. | Page 9-10 |
|  | 20d | Present results of all sensitivity analyses conducted to assess the robustness of the synthesized results. | Page 9-10 |
| **Reporting biases** | 21 | Present assessments of risk of bias due to missing results (arising from reporting biases) for each synthesis assessed. | Page 10 |
| **Certainty of evidence** | 22 | Present assessments of certainty (or confidence) in the body of evidence for each outcome assessed. | Table 2 |
| **DISCUSSION** |  |  |  |
| **Discussion** | 23a | Provide a general interpretation of the results in the context of other evidence. | Page 12 |
|  | 23b | Discuss any limitations of the evidence included in the review. | Page 13-14 |
|  | 23c | Discuss any limitations of the review processes used. | Page 13-14 |
|  | 23d | Discuss implications of the results for practice, policy, and future research. | Page 12-14 |
| **OTHER INFORMATION** |  |  |  |
| **Registration and protocol** | 24a | Provide registration information for the review, including register name and registration number, or state that the review was not registered. | Page 3 |
|  | 24b | Indicate where the review protocol can be accessed, or state that a protocol was not prepared. | Page 14 |
|  | 24c | Describe and explain any amendments to information provided at registration or in the protocol. | Not applicable |
| **Support** | 25 | Describe sources of financial or non-financial support for the review, and the role of the funders or sponsors in the review. | Page 14-15 |
| **Competing interests** | 26 | Declare any competing interests of review authors. | Page 14 |
| **Availability of data, code and other materials** | 27 | Report which of the following are publicly available and where they can be found: template data collection forms; data extracted from included studies; data used for all analyses; analytic code; any other materials used in the review. | Page 14  Supplementary material |

**PRIMSA Abstract Checklist**

| **Topic** | **No.** | **Item** | **Reported?** |
| --- | --- | --- | --- |
| **TITLE** |  |  |  |
| **Title** | 1 | Identify the report as a systematic review. | Yes |
| **BACKGROUND** |  |  |  |
| **Objectives** | 2 | Provide an explicit statement of the main objective(s) or question(s) the review addresses. | Yes |
| **METHODS** |  |  |  |
| **Eligibility criteria** | 3 | Specify the inclusion and exclusion criteria for the review. | Yes |
| **Information sources** | 4 | Specify the information sources (e.g. databases, registers) used to identify studies and the date when each was last searched. | Yes |
| **Risk of bias** | 5 | Specify the methods used to assess risk of bias in the included studies. | Yes |
| **Synthesis of results** | 6 | Specify the methods used to present and synthesize results. | Yes |
| **RESULTS** |  |  |  |
| **Included studies** | 7 | Give the total number of included studies and participants and summarise relevant characteristics of studies. | Yes |
| **Synthesis of results** | 8 | Present results for main outcomes, preferably indicating the number of included studies and participants for each. If meta-analysis was done, report the summary estimate and confidence/credible interval. If comparing groups, indicate the direction of the effect (i.e. which group is favoured). | Yes |
| **DISCUSSION** |  |  |  |
| **Limitations of evidence** | 9 | Provide a brief summary of the limitations of the evidence included in the review (e.g. study risk of bias, inconsistency and imprecision). | Yes |
| **Interpretation** | 10 | Provide a general interpretation of the results and important implications. | Yes |
| **OTHER** |  |  |  |
| **Funding** | 11 | Specify the primary source of funding for the review. | No |
| **Registration** | 12 | Provide the register name and registration number. | Yes |

*From:* Page MJ, McKenzie JE, Bossuyt PM, Boutron I, Hoffmann TC, Mulrow CD, et al. The PRISMA 2020 statement: an updated guideline for reporting systematic reviews. MetaArXiv. 2020, September 14. DOI: 10.31222/osf.io/v7gm2. For more information, visit: [www.prisma-statement.org](file:///C:\\Users\\ssk\\Downloads\\www.prisma-statement.org)

**Supplementary Table S2.** Search strategy

| **Database** | PubMed |
| --- | --- |
| **Search Terms** | Title/Abstract (Fu's subcutaneous needling OR FSN) AND (Knee Osteoarthritis OR Osteoarthritis of the Knee OR Knee Osteoarthritides OR Osteoarthritis of Knee) AND (randomized controlled trial OR controlled clinical trial OR randomized OR randomly OR trial OR RCT) |

| **Database** | Ovid |
| --- | --- |
| **Search Terms** | ((Fu's subcutaneous needling OR FSN) and (Knee Osteoarthritis OR Osteoarthritis of the Knee OR Knee Osteoarthritides OR Osteoarthritis of Knee) and (randomized controlled trial OR controlled clinical trial OR randomized OR randomly OR trial OR RCT)).af. |

| **Database** | EMBASE |
| --- | --- |
| **Search Terms** | ('Fu's subcutaneous needling' OR 'Fu's subcutaneous needling'/exp OR 'FSN') AND ('Knee Osteoarthritis'/exp OR 'Knee Osteoarthritis' OR 'Osteoarthritis of the Knee' OR 'Knee Osteoarthritides' OR 'Osteoarthritis of Knee') AND ('randomized controlled trial'/exp OR 'randomized controlled trial' OR 'controlled clinical trial' OR 'randomized' OR 'randomly' OR 'trial' OR 'RCT') |

| **Database** | Cochrane Library |
| --- | --- |
| **Search Terms** | Fu's subcutaneous needling OR FSN in Title Abstract Keyword AND Knee Osteoarthritis OR Osteoarthritis of the Knee OR Knee Osteoarthritides OR Osteoarthritis of Knee in Title Abstract Keyword AND randomized controlled trial OR controlled clinical trial OR randomized OR randomly OR trial OR RCT in Title Abstract Keyword |

| **Database** | China National Knowledge Infrastructure |
| --- | --- |
| **Search Terms** | (浮针) AND (膝骨关节炎 + 膝关节骨关节炎 + 膝骨性关节炎 + 膝关节骨性关节炎 + 膝痛) AND (随机对照试验 + 试验 + 随机 + 临床试验 +对照 + 临床研究) (Title/Abstract/Keyword) |

| **Database** | VIP Database |
| --- | --- |
| **Search Terms** | (膝骨关节炎 OR 膝关节骨关节炎 OR 膝骨性关节炎 OR 膝关节骨性关节炎 OR 膝痛) 与 (浮针) 与 (随机对照试验 OR 试验 OR 随机 OR 对照 OR 临床研究)(Title/Abstract/Keyword) |

| **Database** | Sinomed Database |
| --- | --- |
| **Search Terms** | ( "膝骨关节炎"[全部字段] OR "膝关节骨关节炎"[全部字段] OR "膝骨性关节炎"[全部字段] OR "膝关节骨性关节炎"[全部字段] OR "膝痛"[全部字段]) AND( "浮针"[全部字段]) AND( "随机对照试验"[全部字段] OR "随机"[全部字段] OR "对照"[全部字段] OR "试验"[全部字段] OR "临床研究"[全部字段]) |

| **Database** | WANFANG Medical |
| --- | --- |
| **Search Terms** | (膝骨关节炎 OR 膝关节骨关节炎 OR 膝骨性关节炎 OR 膝关节骨性关节炎 OR 膝痛) AND (浮针)(随机对照试验 OR 试验 OR 随机 OR 对照 OR 临床研究) (任意字段) |

**Supplementary Table 3. Excluded studies and the reason for exclusion.**

| Study  （in Chinese） | Study  （in English） | Reason for exclusion |
| --- | --- | --- |
| 王友清,黄伟,李卓璐,等.浮针结合艾灸循经筋阿是穴治疗膝骨性关节炎的疗效研究[J].成都医学院学报,2024,19(06):1040-1043. | Efficacy of Fu's Subcutaneous Needling Combined with Moxibustion at Ashi Points Along the Tendons in Treating Knee Osteoarthritis | Intervention was complicated |
| 邓业川,赵敏明.温针灸、浮针及针刺治疗膝骨性关节炎临床研究[J].陕西中医,2020,41(10):1496-1499. | Clinical study on the treatment of knee osteoarthritis with warm acupuncture,Fu's subcutaneous needling and acupuncture | Intervention was complicated |
| 王佩光,张小文,麦美斯,等.广义估计方程评估浮针法联合穴位埋线治疗不同分期膝骨关节炎的疗效[J].中国组织工程研究,2025,29(08):1565-1571. | Generalized equation estimation of the therapeutic effect of Fu's subcutaneous needling therapy combined withacupoint embedding on different stages of human knee osteoarthritis | Intervention was complicated |
| 段卫平,吕一品.浮针疗法治疗膝骨关节炎40例[J].上海针灸杂志,2011,30(11):777-778. | 40 cases of knee osteoarthritis treated by Fu's subcutaneous needling therapy | Do not have a control group. |
| 范德辉.浮针配合股四头肌训练治疗膝骨关节炎的临床研究[J].江西中医学院学报,2008,(04):53-55. | Clinical Study on Treating Knee Ostarthritis with Fu's subcutaneous needling and Training of Musculus Quadriceps Fexoris | Do not have a control group. |
| 梁永翠,黄燕,周立志,等.浮针配合关节腔注射治疗膝骨性关节炎疗效观察[J].中国中医药信息杂志,2012,19(08):73-74. | Observation on therapeutic effect of Fu's subcutaneous needling combined with intra-articular injection on knee osteoarthritis | Do not have a control group. |
| 王建国,高铁梅,朱美华.三种疗法在膝骨关节炎治疗中的效果观察[J].安徽医药,2010,14(05):573-574. | Observation on the effect of three therapies in the treatment of knee osteoarthritis | Outcome were not available |
| 王浩宇,孙晓伟. 浮针联合热敏灸治疗膝骨关节炎临床观察[J]. 山西中医,2024,40(12):29-31. | Efficacy observation of Fu's subcutaneous needling therapy and heat-sensitive moxibustion on knee osteoarthritis | Intervention was complicated |
| 张智,张建华. 熏洗方结合浮针治疗寒湿痹阻型膝骨关节炎临床观察[J]. 光明中医,2024,39(24):4991-4994. | Clinical Observation of the Combined Use of Fumigation and Washing Formula with Fu's subcutaneous needling for Knee Osteoarthritis of Cold-Damp Bi Syndrome | Intervention was complicated |
| 王玉文,朱民顺,梁龙,等.浮针疗法联合塞来昔布胶囊口服治疗早中期膝骨关节炎[J].中医正骨,2025,37(01):76-78. | Fu's acupuncture therapy combined with oral application of celecoxib capsules for treatment of early and middle stage knee osteoarthritis | Intervention was complicated |

**Supplementary Appendix S1 GRADE evidence evaluation criteria**

**Factors that may reduce the level of quality of evidence:**

Each following question had 3 answers: “No serious risk”, “Serious risk” and “Very serious risk”.

Risk of Bias: “Serious risk” when about half of the studies had the lack of randomized methods, allocation concealment methods, and blinded evaluation.“Very serious risk” when almost all the studies had the lack of randomized methods, allocation concealment methods, and blinded evaluation.“No serious risk” when a few or none of the studies had the lack of randomized methods, allocation concealment methods, and blinded evaluation. Low-quality articles had a weak influence on the weight of the total combined effect

Inconsistency: “No serious risk” when I^2^≤50%; “Serious risk” when 50<I^2^≤75%; “Very serious risk” when I^2^>75%

Indirectness: According to PICO principle and Comprehensive consideration in combination with information.

Imprecision: “No serious risk” when the confidence intervals for the effect-size estimates were sufficiently narrow. “Very serious risk” when the confidence intervals for the effect-size estimates were wide. “Serious risk” when the widths of the confidence intervals for the effect-size estimates were in between.

Publication bias: “No serious risk” when Egger’s test P>0.05; “Serious risk”when 0.01<P≤0.05; “Very serious risk” when P≤0.01.

**Factors that may increase the level of quality of evidence:**

Plausible Confounding had 2 answers: “No” and “Yes”. It would be assessed by comprehensive consideration in combination with information.

Magnitude of effect had 3 answers: “No”, “Yes” and “Extremely” related to odds ratio (OR). “No” when 0.5<OR<2; “Yes” when 0.2<OR≤0.5 or 2≤OR<5; “Extremely” when OR≤0.2 or OR≥5.

There were 4 levels of quality: “High”, “Moderate”, “Low” and “Very low”. Evidence of RCTs were initially assessed as “High”. “Serious risk” would reduce 1 level of quality and “Very serious risk” would reduce 2 levels of quality. While “Yes” could promote 1 level of quality and “Extremely” could promote 2 level of quality.

**Supplementary Appendix S2 . Egger’s test results**

Total efficacy rate


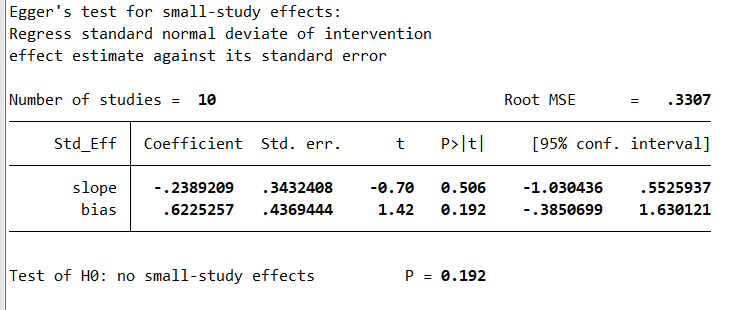


VAS：


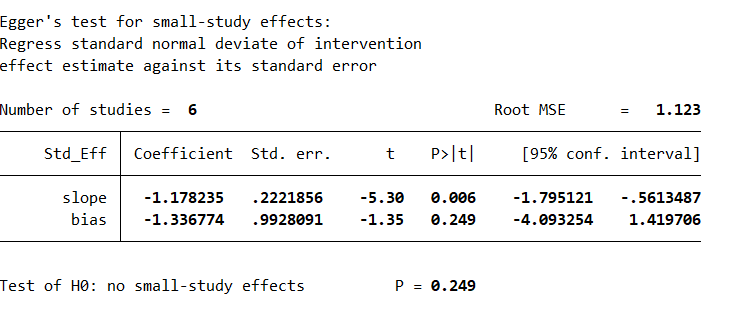


WOMAC：（Liu et al., 2020 weight0%）


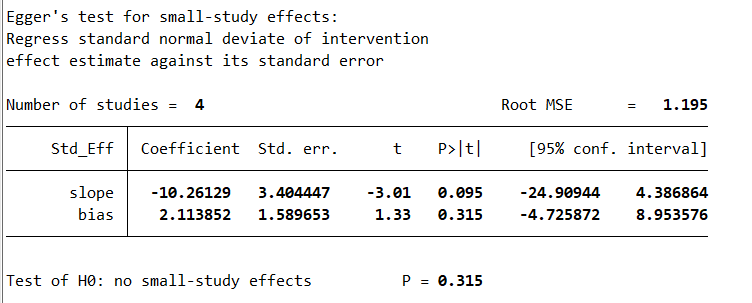


IL-6：


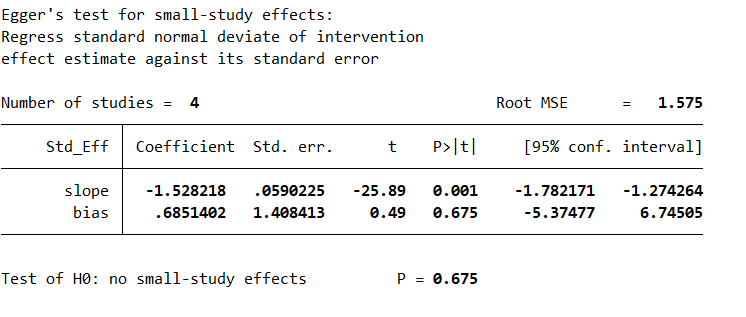


TNF：


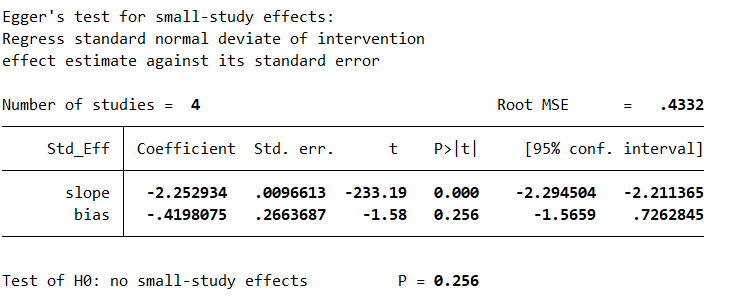


Adverse events： （Chen et al., 2023 weight0%; Zhanget al., 2020 weight0%）

egger's test could not be performed because of insufficient study


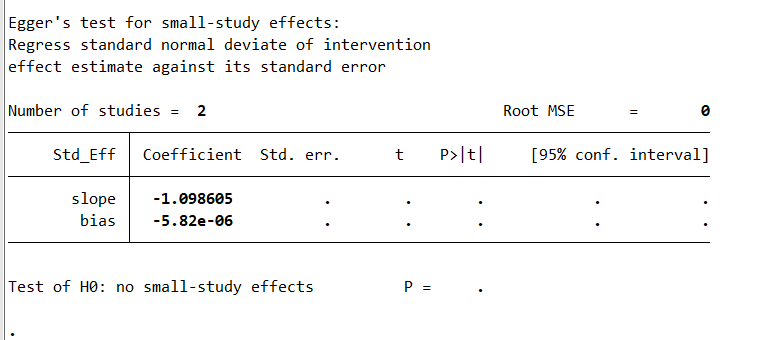

Supplement: Supplementary file 1 [file Table_1.docx]
